# THBS2 Promotes Prostate Cancer Malignancy via INHBA‐Dependent FAK/PI3K/AKT Signaling Activation

**DOI:** 10.1155/ancp/3503659

**Published:** 2026-01-10

**Authors:** Yifeng Yao, Yiyang Guo, Shoulei Liu, Yaojun Li, Shanmiao Chen

**Affiliations:** ^1^ Department of Urology Surgery, Tongxiang First People’s Hospital (Tongxiang Hospital Affiliated to Hangzhou Medical College), No. 1918, Xiaochang East Road, Tongxiang, 314500, Zhejiang, China

**Keywords:** EMT, FAK/PI3K/AKT signaling pathway, INHBA, prostate cancer, THBS2

## Abstract

Prostate cancer (PCa) is a leading malignancy among men, with a growing incidence and mortality rate worldwide. There is a pressing need to identify novel molecular biomarkers and therapeutic targets to improve prognosis and treatment strategies for PCa. We analyzed thrombospondin 2 (THBS2) expression patterns in PCa using The Cancer Genome Atlas (TCGA) datasets and validated findings in clinical tissues and cell lines. Functional assays, including Cell Counting Kit (CCK)‐8 proliferation, flow cytometry apoptosis, wound healing, and Transwell invasion, were performed on PCa cell lines with altered THBS2 expression. Bioinformatic analyses were conducted to explore THBS2‐related pathways, and protein interactions were examined via STRING and Western blot. THBS2 expression was significantly upregulated in PCa tissues and cell lines and correlated with advanced clinicopathological features and poor prognosis. Overexpression of THBS2 promoted PCa cell proliferation, migration, invasion, and epithelial‐mesenchymal transition (EMT), while inhibiting apoptosis. Mechanistically, THBS2 interacted with INHBA to activate the focal adhesion kinase (FAK)/PI3K/AKT signaling pathway, facilitating malignant progression. INHBA knockdown reversed the oncogenic effects of THBS2, indicating a synergistic interaction between THBS2 and INHBA in PCa pathogenesis. THBS2 acts as an oncogene in PCa by promoting proliferation, invasion, and EMT via the FAK/PI3K/AKT pathway in cooperation with INHBA. These findings highlight THBS2 as a promising biomarker for PCa.

## 1. Introduction

Prostate cancer (PCa) represents a significant public health challenge, ranked as the second most prevalent malignancy among males globally, with 1,467,854 newly confirmed cases reported in 2022 [1]. Within the Chinese demographic context, PCa incidence and mortality rates have exhibited a steady upward trajectory since 2012, coinciding with the implementation of prostate‐specific antigen (PSA)‐based screening protocols and the expanding geriatric population [2]. While early detection initiatives and localized therapeutic interventions demonstrate mortality reduction potential, the asymptomatic progression of PCa frequently culminates in advanced‐stage identification or accelerated transition from localized tumors to metastatic dissemination. Although androgen receptor‐directed therapeutics have substantially enhanced clinical outcomes and 5‐year survival rates, ~30%–40% of patients exhibit intrinsic or acquired resistance to these precision interventions [3–5]. As a result, the discovery and application of prognostic biomarkers for survival and therapeutic outcomes, in conjunction with the development of effective strategies to support personalized therapeutic approaches, remain critical challenges in the advancement of precision medicine for PCa management [6, 7].

Thrombospondins (THBSs) constitute an evolutionarily conserved family of extracellular multidomain glycoproteins with calcium‐binding properties and oligomeric structures, first identified in 1971 through the work of Baenziger and colleagues [8]. Comprising five members (THBS1‐THBS5), these proteins serve vital functions in maintaining structural integrity, facilitating cellular adhesion, and mediating signaling pathways within the extracellular matrix. They coordinate cellular‐matrix communication through receptor binding, thereby orchestrating essential biological functions including cell proliferation, migratory activity, and programmed cell death [9]. THBS2 has been recognized as a tumor‐promoting gene in various cancers, including colorectal cancer, non‐small cell lung cancer, gastric cancer, and breast cancer [10–13]. Additionally, THBS2 protein is present in extracellular vesicles and particles, which serves as a key marker for differentiating tumor tissues from normal tissues [14]. The pathological role of THBS2 in various cancer types establishes it as a potential molecular target for combating disease progression. Nevertheless, the precise biological activities and underlying mechanisms of THBS2 in prostate carcinogenesis warrant further investigation.

In summary, this study primarily centers on elucidating the role and underlying mechanisms of THBS2 in PCa. Through these comprehensive investigations, we aim to offer fresh insights into PCa pathogenesis, ultimately facilitating the identification of innovative biomarkers for clinical applications.

## 2. Materials and Methods

### 2.1. Bioinformatic Analysis

RNA‐seq gene expression profiles and associated clinical information were extracted from The Cancer Genome Atlas (TCGA; https://portal.gdc.cancer.gov/). Statistical analyses examining the associations between THBS2 and T stage, N stage, age, and Gleason score were performed in R using the "stats" and "car" packages. Survival analysis was conducted by generating Kaplan–Meier curves with the ’survival’ package in R. Functional annotation, including Gene Ontology (GO), Kyoto Encyclopedia of Genes and Genomes (KEGG) pathway analysis, and Gene Set Enrichment Analysis (GSEA), was completed using the clusterProfiler package. Protein–protein interaction (PPI) networks involving THBS2 were predicted with the STRING database (https://string-db.org/). The bioinformatics analysis was performed using R software, Version 4.2.1.

### 2.2. Specimen Collection

This study employed tissue samples collected from 28 PCa patients who underwent surgical procedures at the Department of Urology, Tongxiang First People’s Hospital, between October 2022 and October 2024. Both PCa tissues and corresponding adjacent normal tissues (collected from a region at least 2 cm away from the tumor margin) were obtained. The accuracy of tissue separation was verified by histopathological examination: tumor samples were confirmed to contain >70% cancer cells, while adjacent normal samples showed normal glandular architecture without malignancy. Immediately after excision, all specimens were rapidly frozen in liquid nitrogen. The entire study was performed in accordance with the principles outlined in the Declaration of Helsinki and received approval from the Ethics Committee of Tongxiang First People’s Hospital.

### 2.3. Cell Culture and Reagents

RWPE‐1, HEK‐293T, and three human PCa cell lines (PC3, DU145, 22Rv1, and LNCaP) were sourced from ATCC. RWPE‐1 cells were maintained in Keratinocyte Serum‐Free Medium (K‐SFM) supplemented with bovine pituitary extract and human epidermal growth factor, incubated at 37°C in an atmosphere containing 5% CO_2_. HEK‐293T cells were grown in Dulbecco’s modified eagle medium (DMEM). PC3, DU145, 22Rv1, and LNCaP cells were cultured in RPMI‐1640 medium (Gibco, USA), and supplemented with 10% fetal bovine serum (FBS; Gibco, USA) and 1% penicillin/streptomycin (Beyotime, China). All cell lines were routinely tested for mycoplasma contamination and maintained at low passage numbers (<25). Cells were subcultured at 80%–90% confluence using 0.25% trypsin‐EDTA (Gibco, USA). The focal adhesion kinase (FAK) inhibitor Y15 was obtained from MedChemExpress (MCE, China).

### 2.4. Cell Transfection and Stable Cell Line Establishment

Lentiviral constructs for overexpression (OE THBS2 and OE INHBA), utilizing the expression vector and shRNA‐mediated knockdown vectors (shTHBS2 and shINHBA) together with their respective control vectors (OECtrl and shCtrl), were obtained from VectorBuilder (Guangzhou, China). The lentiviral preparations had a titer of 1 × 10^9^ TU/mL. PC3 cells were transduced with these viral particles at a multiplicity of infection (MOI) of 5 for a duration of 48 h. Following infection, stable cell lines were selected using 2 μg/mL puromycin (Sigma, USA) for 14 days, with media replaced every 3 days. Successful transfection was quantitatively confirmed via quantitative real‐time PCR (qRT‐PCR) (for mRNA) and Western blotting (WB) (for protein) after three passages post‐selection. The target sequences of the shRNAs were as follows: THBS2, CCCTCCTAAGACAAGGAACAT; INHBA, GCTTCTGAACGCGATCAGAAA.

### 2.5. WB

Proteins were extracted from both tissues and cells using RIPA buffer containing 1 mM PMSF (Solarbio, China). Protein concentrations were assessed using the BCA assay kit (Solarbio, China). Equal amounts of protein were loaded onto 10% SDS‐PAGE gels for electrophoretic separation and subsequently transferred onto PVDF membranes. The membranes were blocked with skim milk dissolved in TBST for 1 h, and then incubated overnight at 4°C with primary antibodies specific to the proteins of interest. The following primary antibodies (all from Abcam, UK) were used: Vimentin (ab92547, 1:2000), THBS2 (ab112543, 1:1000), INHBA (ab97705, 1:1000), FAK (ab40794, 1:1000), p‐FAK (ab81298, 1:1000), PI3K (ab302958, 1:1000), p‐PI3K (ab191606, 1:1000), AKT (ab8933, 1:1000), p‐AKT (ab38449, 1:1000), E‐cadherin (ab40772, 1:1000), N‐cadherin (ab18203, 1:1000), and GAPDH (ab8245, 1:2000). After primary antibody incubation, membranes were washed and then exposed to HRP‐conjugated secondary antibodies for 1 h at room temperature. Signal detection was performed using the ECL chemiluminescence system (Millipore, USA).

### 2.6. RNA Extraction and qRT‐PCR

Total RNA was isolated from both tissue samples and cells using Trizol reagent (TaKaRa, Japan). cDNA was synthesized from the extracted RNA using the cDNA Synthesis Kit (Bio‐Rad, USA) according to the manufacturer’s instructions. Quantitative PCR was then performed with Universal SYBR Green Supermix (Bio‐Rad, USA) on a CFX96 Real‐Time PCR System (Bio‐Rad, USA). GAPDH served as the internal control for normalizing mRNA expression levels. The primers utilized were as follows:

THBS2: Forward 5′‐GGACTTCAGTGGCACATTCT‐3′, Reverse 5′‐TGCTTCCACATCACCACATAG‐3′
INHBA: Forward 5′‐TCTGCAGTAGTGTGGACTAGAA‐3′, Reverse 5′‐CCTGGGTAATTGGGTAGGAAAG‐3′
GAPDH: Forward 5′‐CACCCACTCCTCCACCTTTG‐3′, Reverse 5′‐CCACCACCCTGTTGCTGTAG‐3′

### 2.7. Proliferation Assay

PC3 cells subjected to various treatments were resuspended in 100 μL of culture medium and plated in 96‐well plates at a density of 5000 cells per well. The cells were then incubated for specified time points (1–4 days). Subsequently, 10 μL of Cell Counting Kit‐8 (CCK8; Boster, USA) solution was added to each well, followed by a further 2‐h incubation in a cell culture incubator. Cell proliferation was assessed by measuring the absorbance at 450 nm with a microplate reader.

### 2.8. Cell Apoptosis Assay

Cell apoptosis was evaluated using flow cytometry following standard procedures. Cells were seeded in 6‐well plates at a density of 1 × 10^6^ cells per well and harvested after incubation. After being washed twice with PBS, apoptosis was analyzed with the Annexin V‐FITC/PI apoptosis detection kit (Keygen Biotech, China) using a CytoFLEX flow cytometer (Beckman Coulter, USA).

### 2.9. Wound Healing Assay

Cells were plated in 6‐well plates at a concentration of 1 × 10^6^ cells per well and maintained in DMEM lacking FBS for 24 h. Linear scratches were created using a 100 μL plastic pipette tip to simulate wounds. Images of the scratch areas were taken immediately and again after 24 h. Cell‐free regions and the extent of wound closure were quantified by analyzing these digital images.

### 2.10. Transwell Assay

For the invasion assay, 24‐well Transwell chambers with 8 μm pore size inserts pre‐coated with Matrigel were used. Cells (1 × 10^5^) suspended in SFM were placed in the upper chamber, while the lower chamber contained medium supplemented with 10% FBS. After 24 h of incubation, non‐invading cells were removed from the upper side of the membrane. The cells that had migrated to the underside were fixed and stained with 0.1% crystal violet, and then evaluated.

### 2.11. Co‐Immunoprecipitation (Co‐IP) Assay

Exogenous Co‐IP: HEK‐293T cells were co‐transfected with Flag‐THBS2 and HA‐INHBA constructs. After 48 h, cells were lysed in IP buffer (20 mM Tris‐HCl [pH 7.4], 150 mM NaCl, 1 mM EDTA [pH 8.0], 1% NP‐40, 1 × phosphatase inhibitor cocktail [MedChemExpress, USA], and 1 × protease inhibitor cocktail [Roche, Switzerland]) on ice for 30 min. Lysates were centrifuged at 12,000×*g* (4°C, 10 min), and supernatants were incubated with anti‐flag M2 magnetic beads (Sigma, USA) overnight at 4°C. Beads were washed four times with IP buffer, resuspended in 1 × Laemmli buffer (Bio‐Rad, USA), and denatured (95°C, 10 min). Eluted proteins were analyzed by immunoblotting. Endogenous‐like Co‐IP: flag‐THBS2‐transfected PC3 cells were lysed as above. Cleared lysates were incubated with anti‐INHBA antibody (ab97705, Abcam, UK) overnight at 4°C, followed by protein A/G magnetic beads (Thermo Fisher, USA) for 2 h. Normal rabbit IgG (ab172730, Abcam, UK) served as control. Subsequent steps matched the exogenous Co‐IP protocol. Input lysates (10%) were analyzed in parallel.

### 2.12. Statistical Analysis

All statistical analyses were performed using IBM SPSS Statistics version 22.0 (IBM, USA). Intergroup comparisons were conducted through Student’s *t*‐test (two‐group analysis) or one‐way ANOVA (multigroup comparisons), with all experiments independently repeated three times. Results were considered statistically significant when *p* < 0.05. Data are presented as the mean ± standard deviation (SD).

## 3. Results

### 3.1. THBS2 Demonstrates Marked Elevation in PCa

Pan‐cancer analysis of TCGA datasets revealed markedly elevated THBS2 expression in various malignancies relative to matched normal tissues, with PCa also demonstrating prominent upregulation (Figure 1A,B). Elevated THBS2 expression levels showed statistically significant positive correlations with advanced T‐stage, N‐stage, Gleason score, and older age in the TCGA‐PRAD cohort analysis (Figure 1C–F). Kaplan–Meier analysis demonstrated significantly reduced progression‐free interval (PFI) in high‐THBS2 expressors (HR = 1.59, 95% CI: 1.05–2.42; *p* = 0.03) (Figure 1G). WB and qRT‐PCR analyses concurrently confirmed markedly elevated THBS2 expression in PCa tissues relative to adjacent normal tissues (Figure 1H,I). Quantitative assessment using WB and qRT‐PCR revealed consistent upregulation of THBS2 expression in PCa cell lines (22Rv1, LNCaP, DU145, and PC3) compared with nonneoplastic prostate epithelial cells (RWPE‐1) (Figure 1J,K).

Figure 1THBS2 demonstrates marked elevation in PCa. (A) Pan‐cancer analysis of THBS2 transcript levels was conducted using TCGA data. (B) Additional confirmation of THBS2 overexpression in PCa was obtained through independent TCGA‐PRAD dataset validation. (C‐F) Clinical correlations between THBS2 expression and tumor progression parameters (T staging, N staging, Gleason grading) along with age stratification in TCGA‐PRAD cases. (G) Survival curves revealed diminished PFI among high THBS2 expressors in the TCGA‐PRAD cohort. (H,I) WB and qRT‐PCR confirmed enhanced THBS2 protein/mRNA abundance in tumor versus paired normal tissues. (J,K) Cell line validation demonstrated consistent elevation of THBS2 at both translational and transcriptional levels in 22Rv1, LNCaP, DU145, and PC3 cells compared to normal RWPE‐1 epithelial controls.  ^∗^ *p* < 0.05;  ^∗∗^ *p* < 0.01;  ^∗∗∗^ *p* < 0.001.

**Figure:**
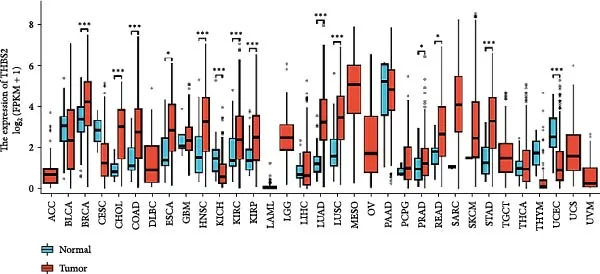
(A)

**Figure:**
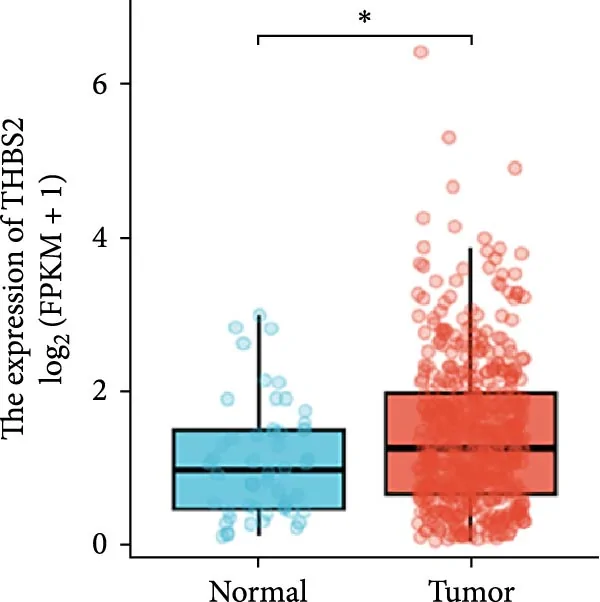
(B)

**Figure:**
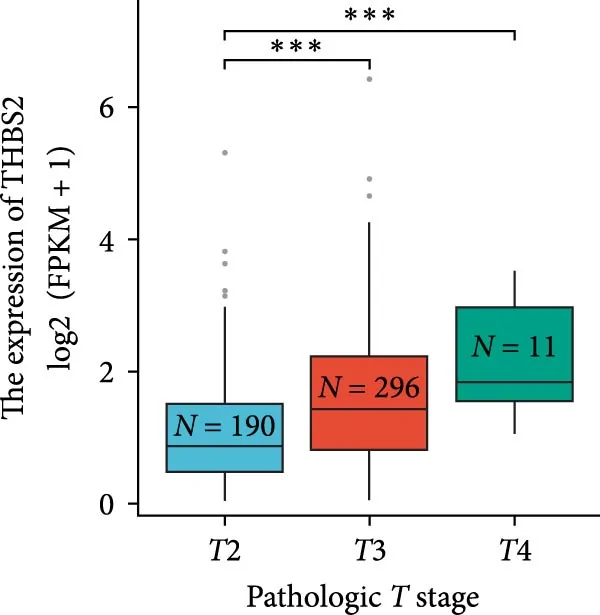
(C)

**Figure:**
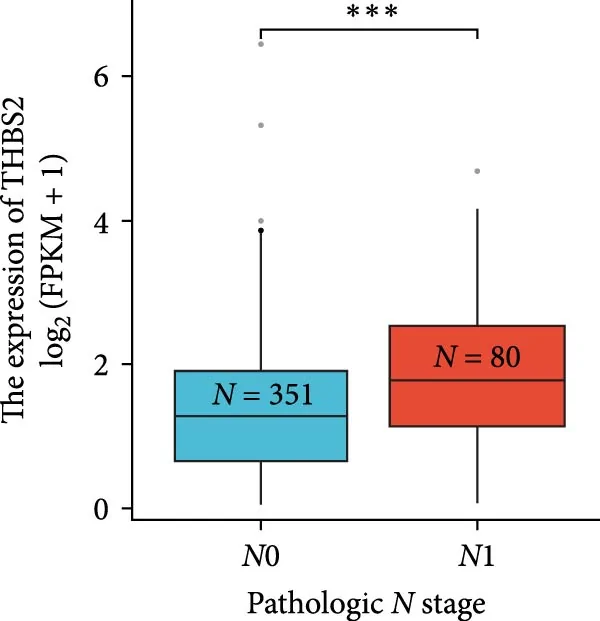
(D)

**Figure:**
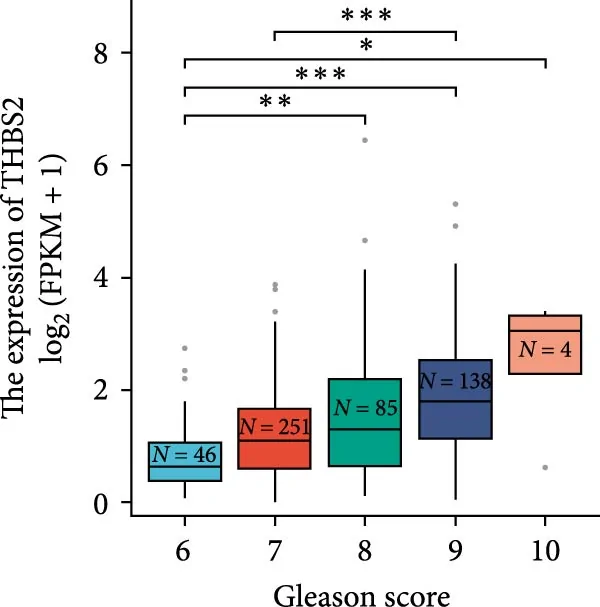
(E)

**Figure:**
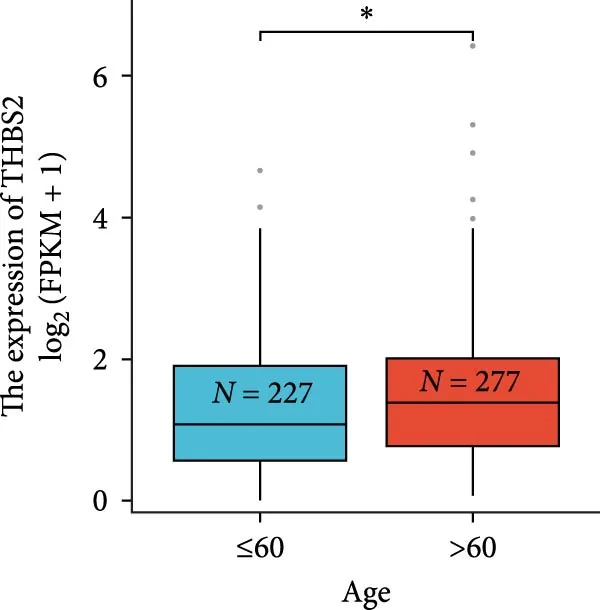
(F)

**Figure:**
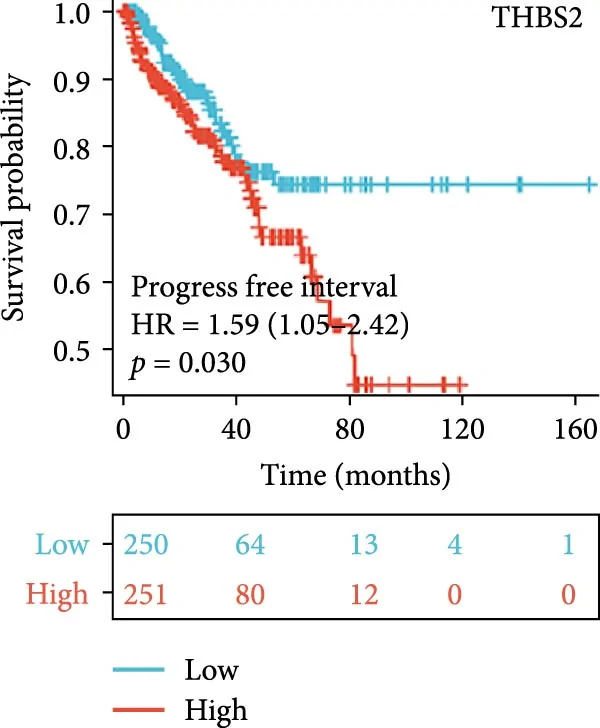
(G)

**Figure:**
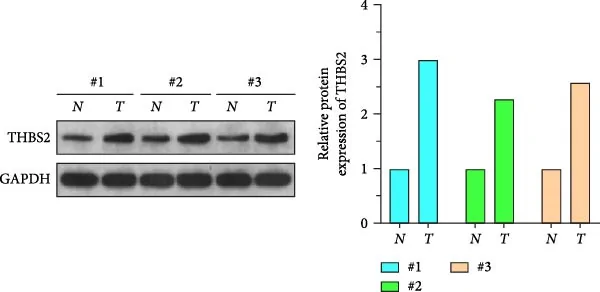
(H)

**Figure:**
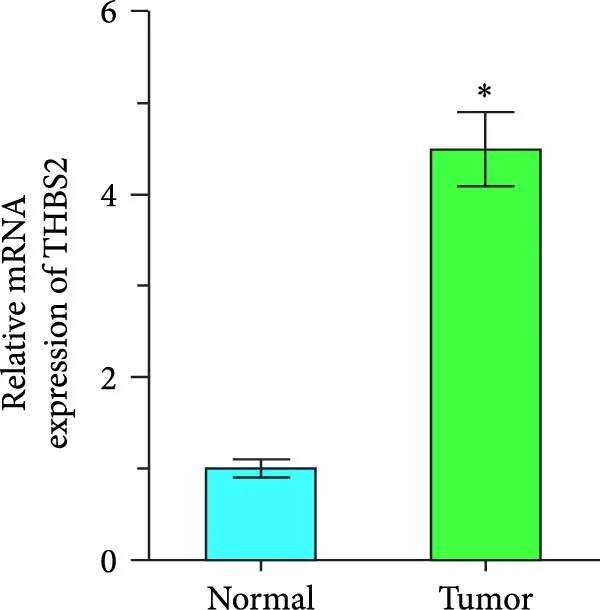
(I)

**Figure:**
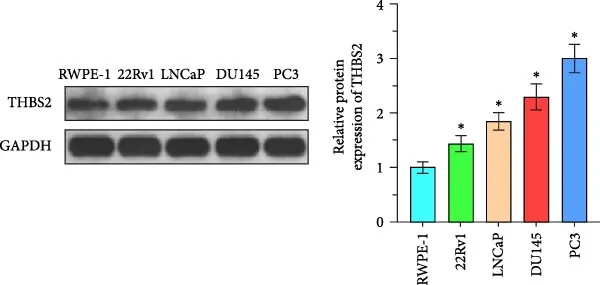
(J)

**Figure:**
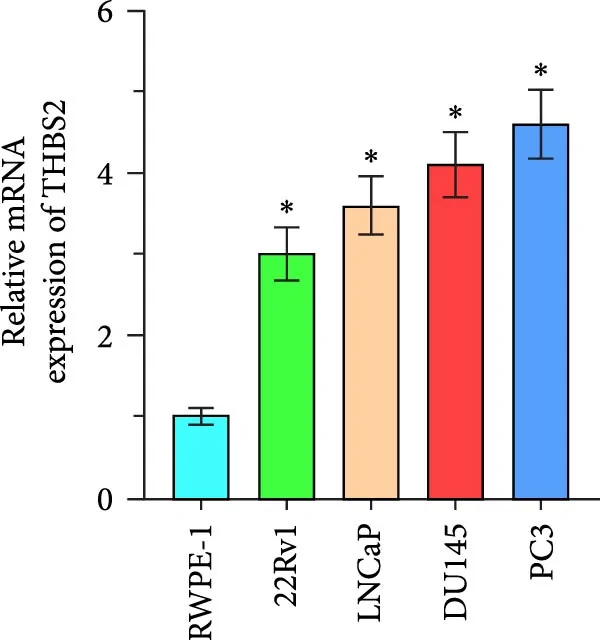
(K)

### 3.2. THBS2 Enhances the Proliferative Potential of PCa Cells While Inhibiting Their Apoptotic Processes

To explore the functional role of THBS2 in PCa, PC3 cell lines with THBS2 overexpression and knockdown were established through lentiviral transduction. Subsequent WB analysis confirmed successful modulation of THBS2 protein expression (Figure 2A). Flow cytometric analysis revealed that enforced THBS2 expression markedly attenuated apoptotic activity in PC3 cells, while, conversely, THBS2 knockdown significantly promoted apoptosis (Figure 2B). CCK‐8 assay demonstrated that THBS2‐overexpressing substantially enhanced PC3 cellular growth kinetics, while, conversely, knockdown of THBS2 expression resulted in significant proliferative inhibition (Figure 2C).

Figure 2THBS2 enhances the proliferative potential of PCa cells while inhibiting their apoptotic processes. (A) The successful generation of THBS2 overexpression and knockdown models in PC3 cells was validated using WB analysis. (B) Cellular proliferation dynamics were quantified via CCK‐8 assays. (C) Flow cytometry was utilized to quantify the apoptotic cell populations in PC3 cells.  ^∗^ *p* < 0.05.

**Figure:**
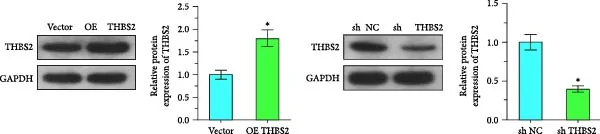
(A)

**Figure:**
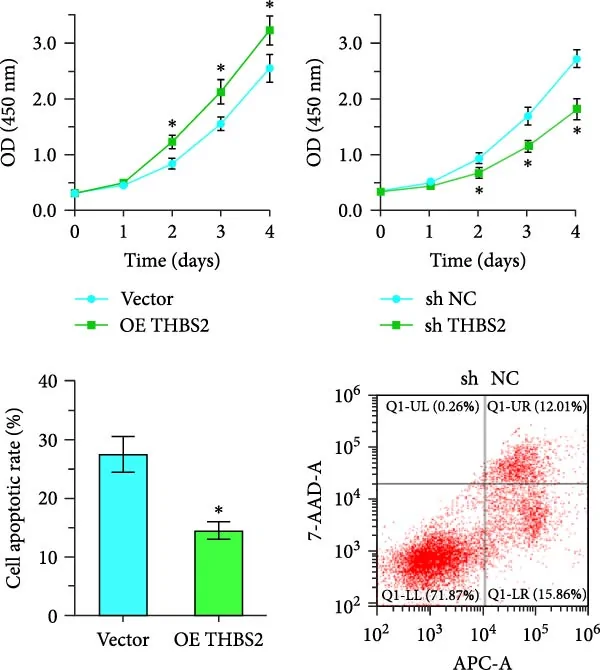
(B)

**Figure:**
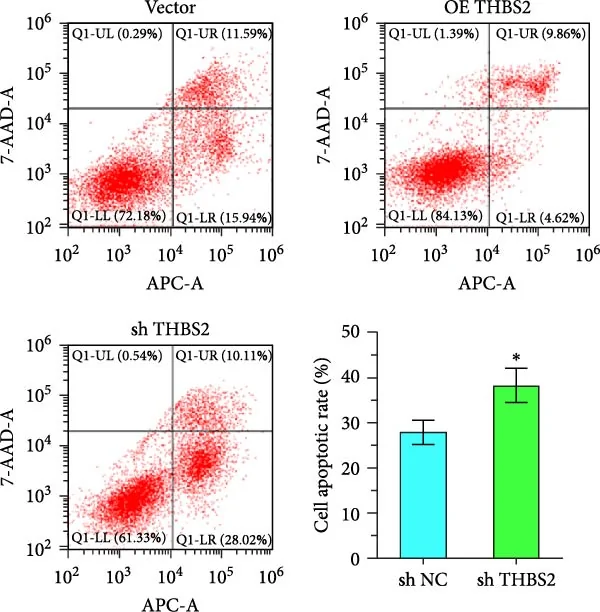
(C)

### 3.3. THBS2 Promotes the Migration and Invasion Capabilities of PCa Cells

The role of THBS2 in regulating metastatic potential was explored in PC3 cells. Wound healing assays indicated that cells overexpressing THBS2 displayed augmented migratory ability, which was substantially diminished upon THBS2 knockdown (Figure 3A). Consistent with these findings, Transwell invasion experiments showed that enforced THBS2 expression enhanced invasive capacity, while downregulation of THBS2 markedly suppressed this process (Figure 3B).

Figure 3THBS2 promotes the migration and invasion capabilities of PCa cells. (A) The migratory activity of PC3 cells was evaluated using a scratch wound healing assay. (B) The invasive capacity of PC3 cells was quantified through Transwell assay.  ^∗^ *p* < 0.05.

**Figure:**
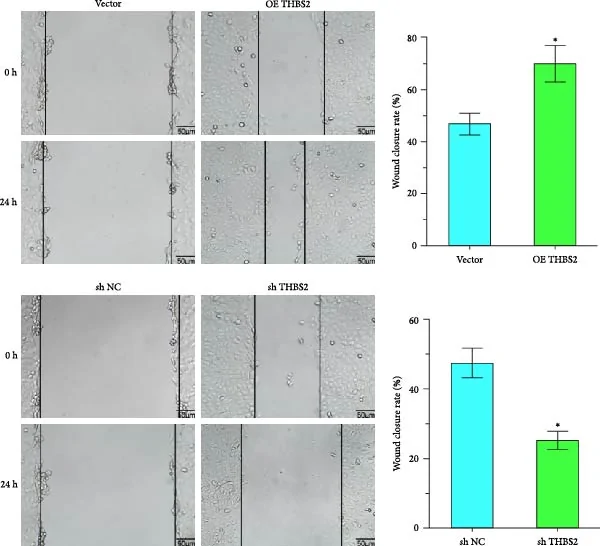
(A)

**Figure:**
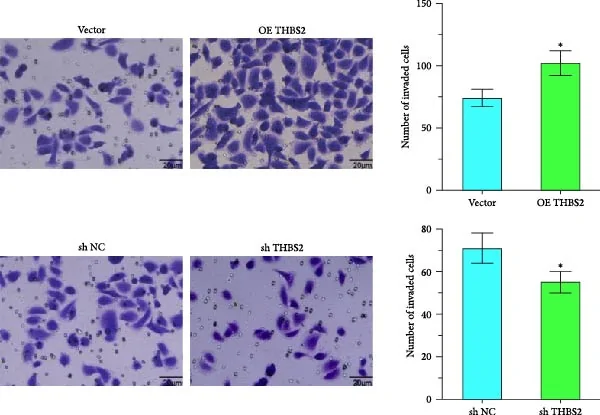
(B)

### 3.4. Identification of THBS2‐Coexpressed Genes and Pathway Enrichment Analysis in PCa

To elucidate the biological mechanisms of THBS2, transcriptomic data from the TCGA‐PRAD cohort were analyzed to identify genes that exhibited significant upregulation and downregulation and were closely correlated with THBS2 (Figure 4A,B). To further investigate these genes, a detailed analysis was conducted using GO and KEGG. The findings from the GO and KEGG analyses indicated that the upregulated genes showed substantial enrichment, especially in processes related to cell–cell adhesion, the PI3K/AKT signaling pathway, and focal adhesion mechanisms (Figure 4C,D). The results from both enrichment analyses indicated that down‐regulated genes exhibited significant enrichment, especially in pathways of neurodegeneration—multiple diseases and response to toxic substances (Figure 4E,F).

Figure 4Identification of THBS2‐coexpressed genes and pathway enrichment analysis in PCa. (A,B) The TCGA‐PRAD dataset was utilized for volcano plot and heatmap analysis to detect genes correlated with THBS2.(C,D)KEGG and GO analyses were conducted on genes positively correlated with THBS2 in PCa. (E,F)KEGG and GO enrichment analyses were performed on genes negatively correlated with THBS2 in PCa.

**Figure:**
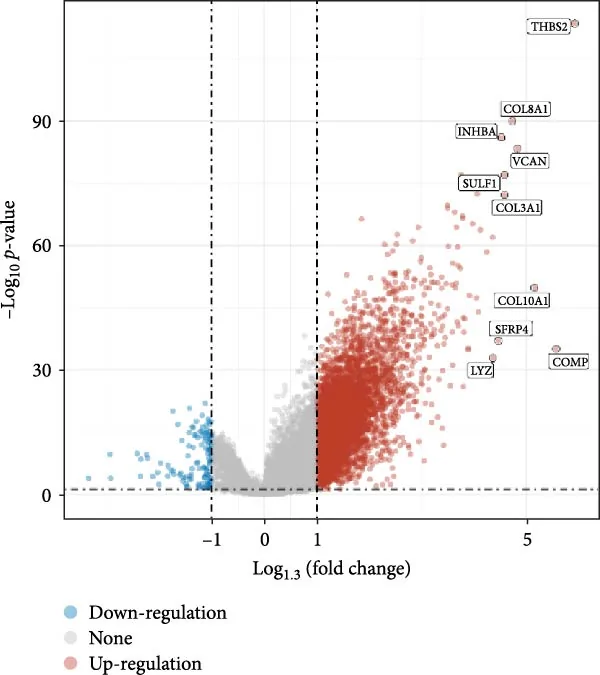
(A)

**Figure:**
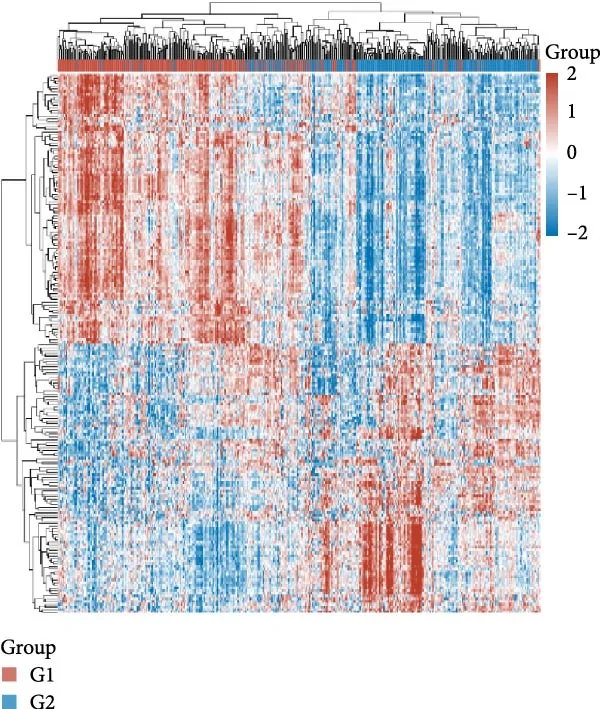
(B)

**Figure:**
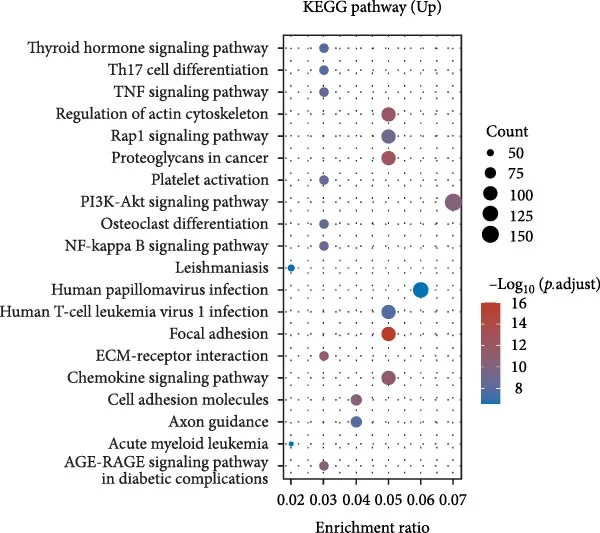
(C)

**Figure:**
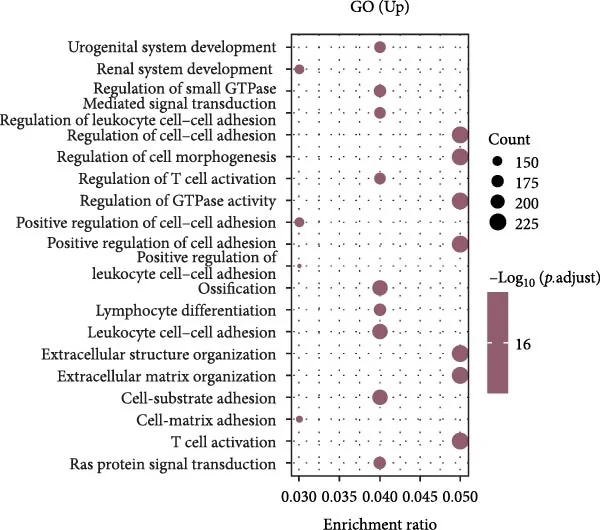
(D)

**Figure:**
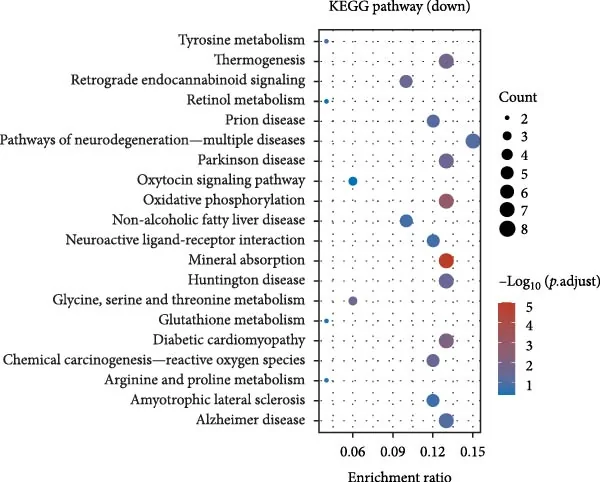
(E)

**Figure:**
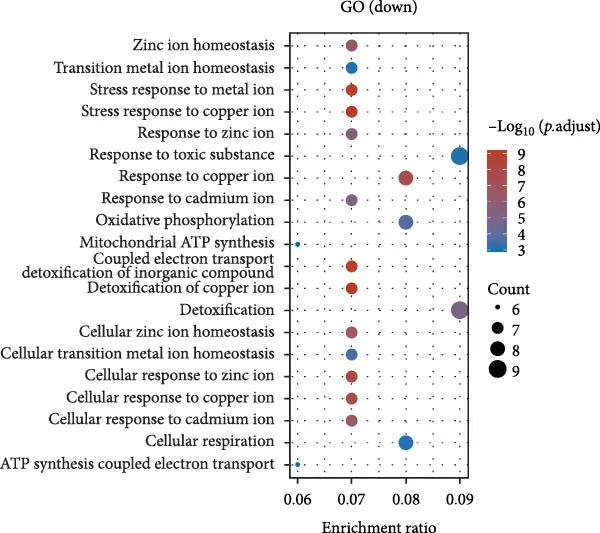
(F)

### 3.5. THBS2 Modulates INHBA Expression in PCa Cells

The TCGA‐PRAD cohort was interrogated to delineate THBS2‐associated co‐expression genes in PCa, elucidating its molecular pathway involvement. Hierarchical clustering analysis of transcriptomic data visualized the 20 most significantly correlated genes through heatmap representation (Figure 5A). Cross‐validation demonstrated strong positive covariation between THBS2 and INHBA expression profiles (Spearman’s *r* = 0.891) (Figure 5B). STRING database interrogation (confidence score = 0.668) further identified a possible interaction e between THBS2 and INHBA proteins (Figure 5C). WB and qRT‐PCR analyses revealed that INHBA expression was markedly higher in PCa tissues compared to adjacent normal tissues (Figure 5D,E). Direct physical interaction between THBS2 and INHBA was validated through two complementary approaches: endogenous Co‐IP in PCa PC3 cells and exogenous Co‐IP in transfected HEK293T cells (Figure 5F,G). Moreover, WB results indicated that upregulation of THBS2 led to an increase in INHBA expression in PC3 cells, whereas THBS2 knockdown resulted in a reduction of INHBA levels within these cells (Figure 5H). To further examine this relationship, lentiviral transduction was utilized to establish stable INHBA overexpression and knockdown in PC3 cells, with subsequent WB confirming the successful modulation of INHBA expression (Figure 5I).

Figure 5THBS2 modulates INHBA expression in PCa cells. (A) Heatmap visualization highlights the top 20 THBS2‐coexpressed genes in TCGA‐PRAD. (B) Significant positive correlation between INHBA and THBS2 expression was observed (Spearman’s rho = 0.891). (C) STRING database PPI analysis confirmed THBS2‐INHBA molecular interaction. (D,E) Comparative quantification of INHBA expression in PCa versus adjacent normal tissues was achieved through WB and qRT‐PCR. (F) Endogenous Co‐IPwas performed in PC3 cells to confirm physical interaction between natively expressed THBS2 and INHBA proteins. (G) Exogenous Co‐IPin transfected HEK293T cells validated THBS2‐INHBA binding using epitope‐tagged constructs. (H) THBS2 overexpression/knockdown in PC3 cells reciprocally altered INHBA protein abundance as evidenced by WB quantification. (I) The successful generation of INHBA overexpression and knockdown models in PC3 cells was validated using WB analysis.  ^∗^ *p* < 0.05;  ^∗∗∗^ *p* < 0.001.

**Figure:**
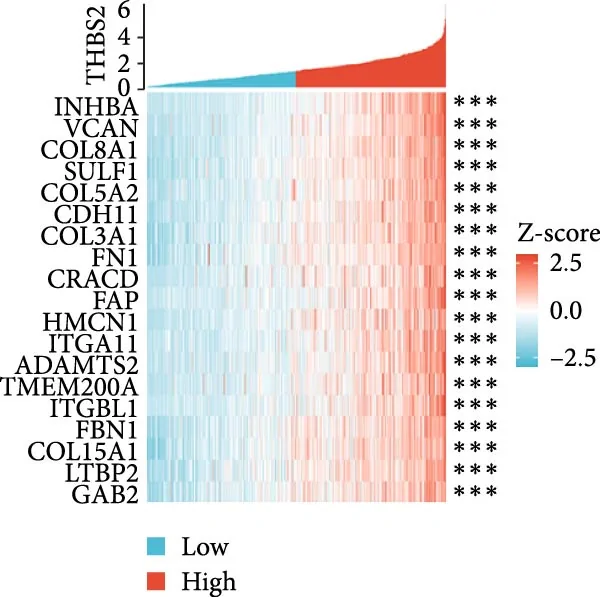
(A)

**Figure:**
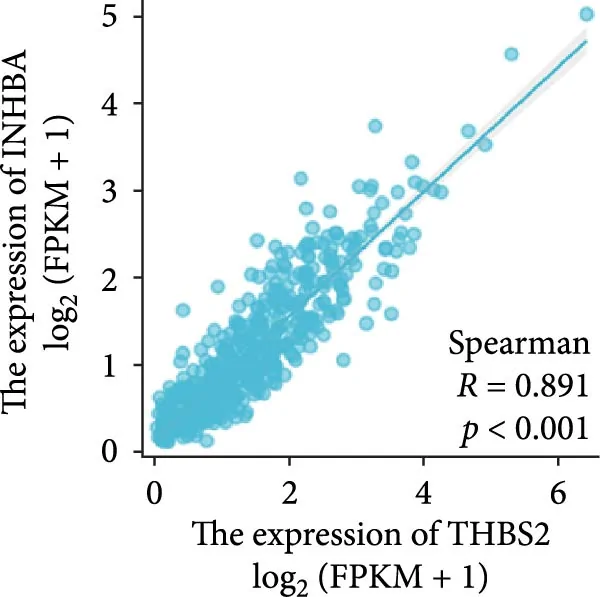
(B)

**Figure:**
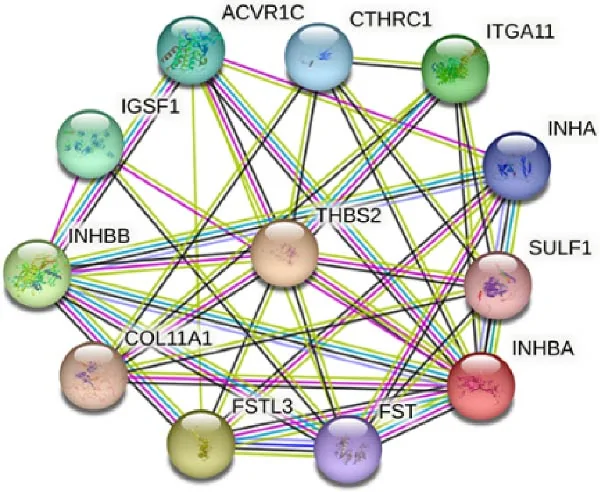
(C)

**Figure:**
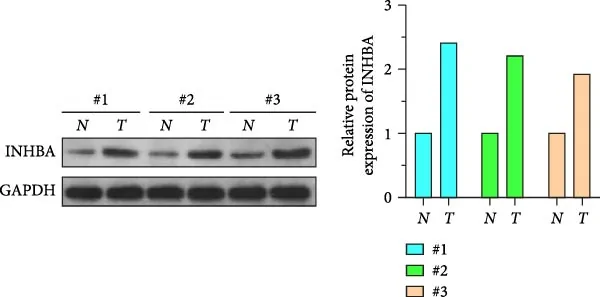
(D)

**Figure:**
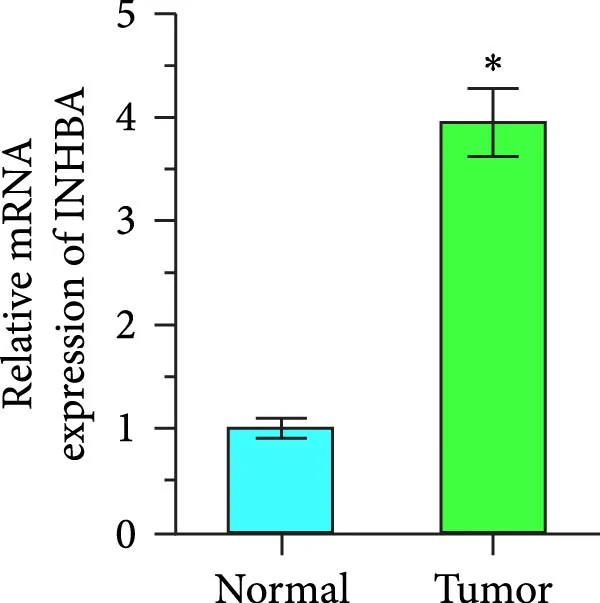
(E)

**Figure:**
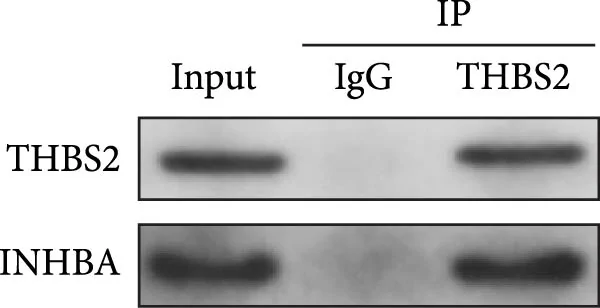
(F)

**Figure:**
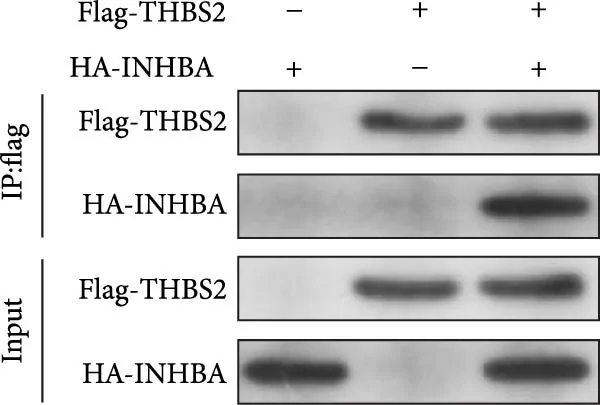
(G)

**Figure:**
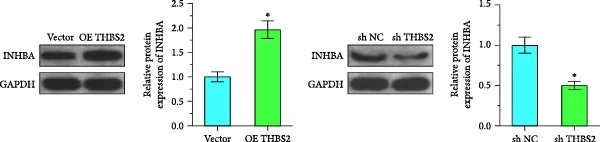
(H)

**Figure:**
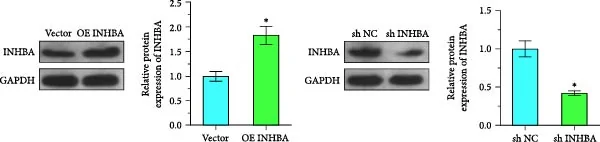
(I)

### 3.6. THBS2 Synergistically Interacts With INHBA to Promote Malignant Progression in PCa

Transwell invasion assays revealed that INHBA knockdown significantly abrogated the THBS2‐overexpression‐mediated enhancement of cellular invasiveness, whereas INHBA overexpression restored the invasive capacity diminished by THBS2 suppression (Figure 6A). Consistently, CCK‐8 proliferation assays indicated that THBS2 upregulation facilitated tumor cell growth, which was counteracted by INHBA downregulation. Conversely, THBS2 depletion suppressed proliferative activity, an effect reversed upon INHBA upregulation (Figure 6B).

Figure 6THBS2 synergistically interacts with INHBA to promote malignant progression in PCa. (A) Cellular invasion capacity of transfected PC3 cells was quantified using Transwell analysis. (B) Proliferation of transfected PC3 cells were determined through CCK‐8 viability assays.  ^∗^ *p* < 0.05.

**Figure:**
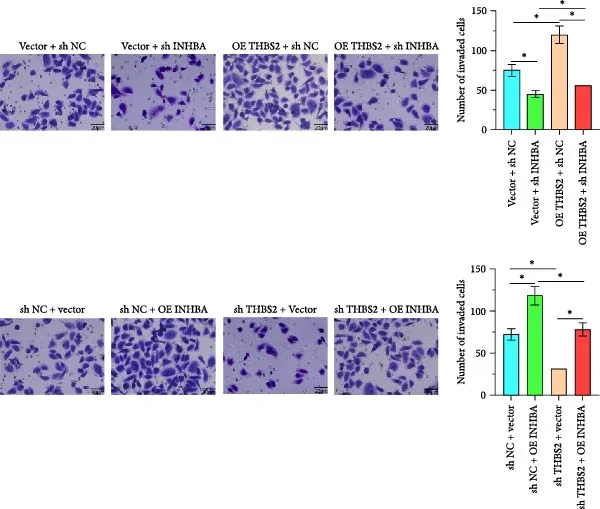
(A)

**Figure:**
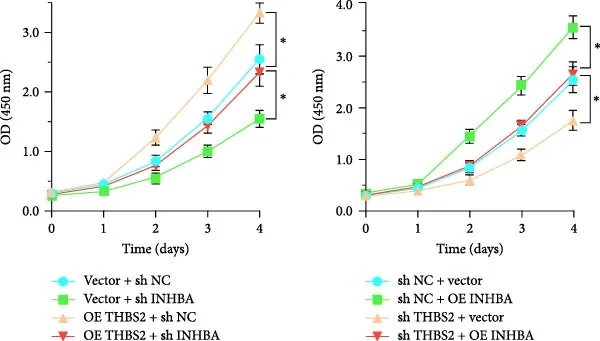
(B)

### 3.7. THBS2 Modulates Epithelial‐Mesenchymal Transition (EMT) Progression in PCa Through FAK/PI3K/AKT Signaling Axis via Molecular Interaction With INHBA

Bioinformatic analysis of the TCGA‐PRAD cohort using GSEA demonstrated pronounced activation of focal adhesion, PI3K‐AKT signaling cascade, and EMT processes in PCa specimens exhibiting elevated THBS2 expression (Figure 7A–C). Based on integrated findings from GO, KEGG, and GSEA analyses, we performed WB assays in PCa to investigate the regulatory effects of the THBS2‐INHBA axis on FAK‐PI3K‐AKT signaling and EMT. WB analysis demonstrated that INHBA inhibition significantly attenuated THBS2‐induced upregulation of phosphorylated FAK, PI3K, AKT, and mesenchymal markers Vimentin/N‐cadherin, while restoring E‐cadherin expression (Figure 7D). Conversely, INHBA overexpression reversed the THBS2 suppression‐induced downregulation of phosphorylated FAK, PI3K, AKT, and mesenchymal markers, along with the elevation of E‐cadherin expression caused by THBS2 inhibition (Figure 7E).

Figure 7THBS2 modulates EMT progression in PCa through FAK/PI3K/AKT signaling axis via molecular interaction with INHBA. (A–C) Gene set enrichment analysis (GSEA) revealed differential activation of focal adhesion (NES = 2.316, FDR < 0.001), PI3K/AKT pathway (NES = 2.213, FDR < 0.001), and EMT‐related genes (NES = 2.753, FDR < 0.001) in samples stratified by THBS2 expression levels. (D,E) Comparative analysis of FAK (p‐FAK), PI3K (p‐PI3K), AKT (p‐AKT) and EMT markers (E‐cadherin, Vimentin, N‐cadherin) demonstrated pathway modulation in PC3 cells.  ^∗^ *p* < 0.05.

**Figure:**
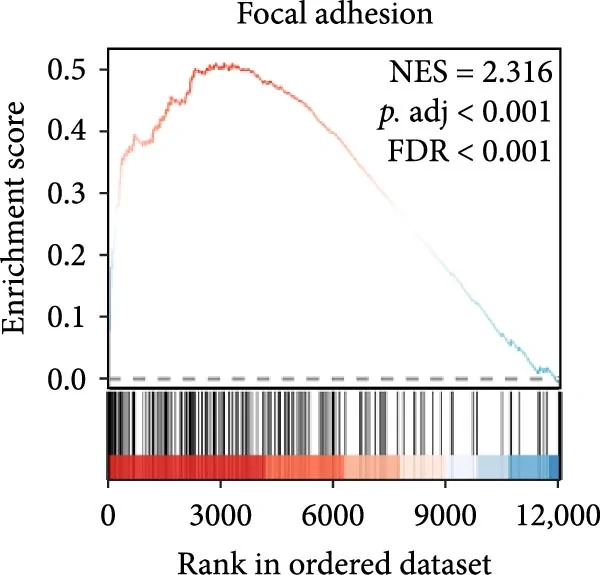
(A)

**Figure:**
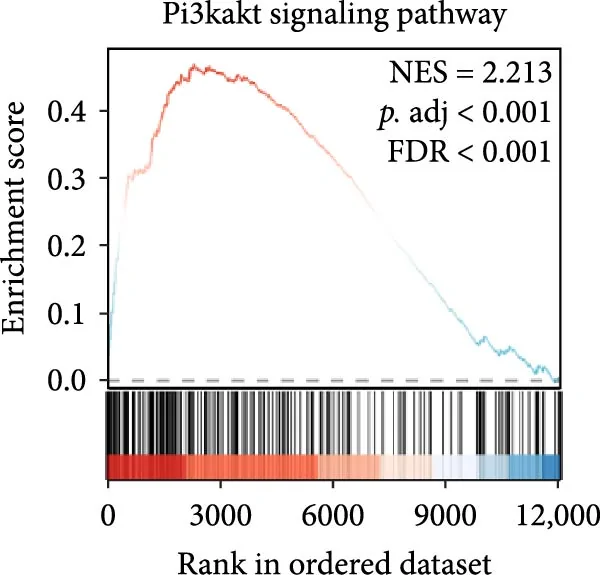
(B)

**Figure:**
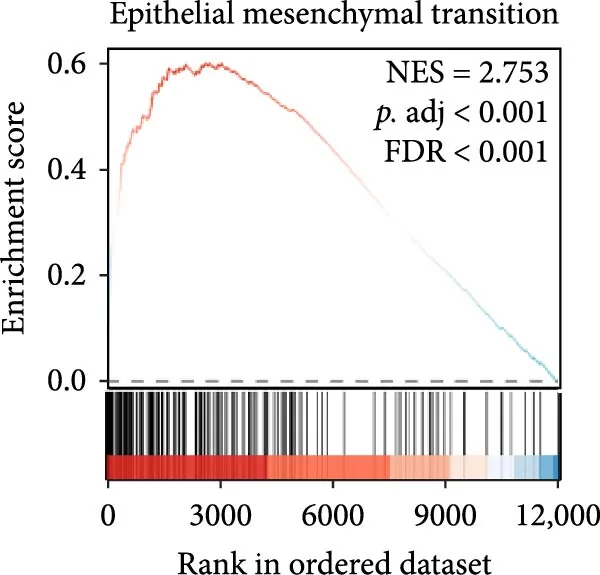
(C)

**Figure:**
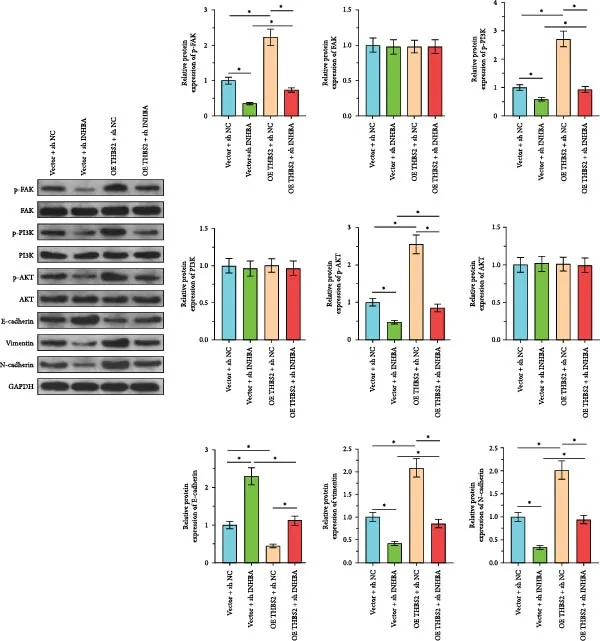
(D)

**Figure:**
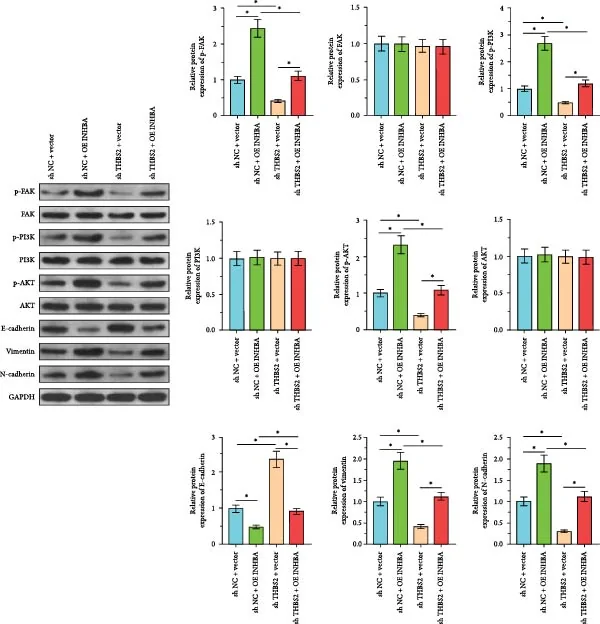
(E)

### 3.8. The FAK Inhibitor Y15 Reverses THBS2‐Driven EMT and malignant Progression in PCa by Blocking the FAK/PI3K/AKT Pathway

To confirm the functional dependency of THBS2 on FAK signaling, we treated THBS2‐overexpressing PC3 cells with the specific FAK inhibitor Y15 (10 µM). Y15 treatment effectively blocked the THBS2‐induced activation of the FAK/PI3K/AKT pathway and the associated EMT (Figure 8A). Consequently, the enhanced invasive and proliferative capacities resulting from THBS2 overexpression were significantly suppressed by FAK inhibition (Figure 8B,C). These results demonstrate that the pro‐tumorigenic effects of THBS2 are critically dependent on FAK signaling.

Figure 8The FAK inhibitor Y15 reverses THBS2‐driven EMT and malignant progression in PCa by blocking the FAK/PI3K/AKT pathway. (A) WB analysis of signaling and EMT markers in PC3 cells under indicated conditions. THBS2 overexpression activated FAK/PI3K/AKT signaling and induced EMT, effects which were blocked by Y15. (B,C) Rescue functional assays showing that Y15 treatment nullifies the invasive and proliferative advantages conferred by THBS2 overexpression. ^∗^ *p* < 0.05.

**Figure:**
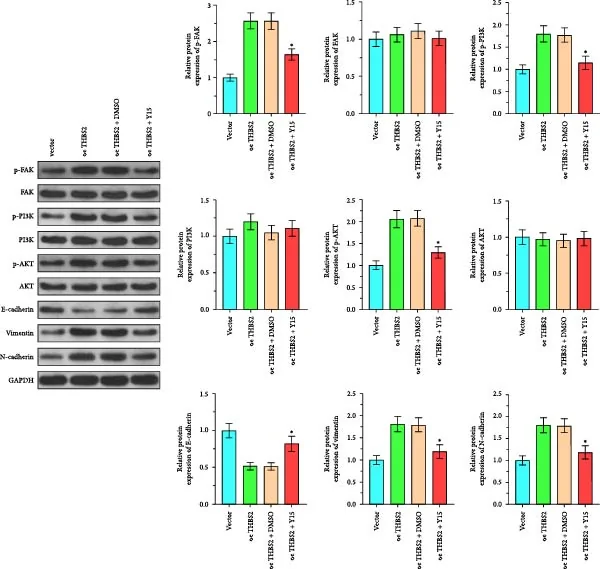
(A)

**Figure:**
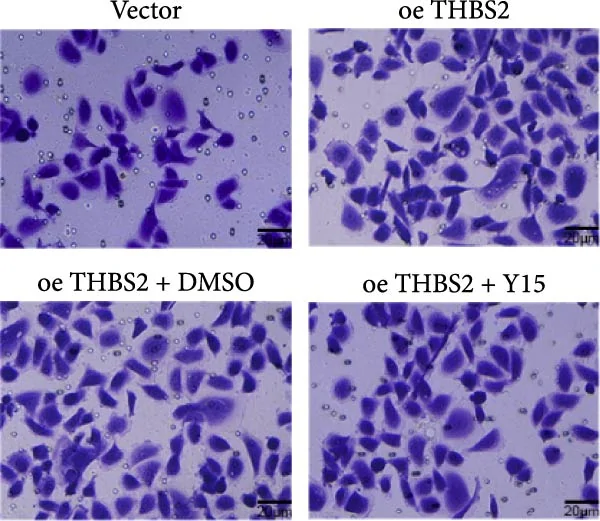
(B)

**Figure:**
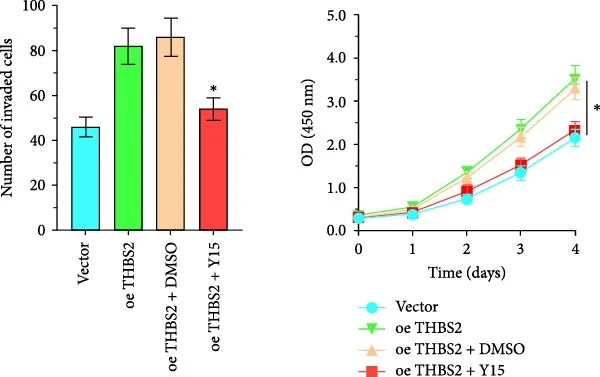
(C)

## 4. Discussion

PCa is marked by uncontrolled cellular proliferation within the prostate gland. Mortality associated with PCa primarily results from metastatic spread, where cancer cells migrate to distant sites such as lymph nodes, the spine, and other vital organs [7]. Addressing PCa therapeutically, particularly in cases involving metastasis, remains challenging due to limited insights into the molecular mechanisms that fuel cancer progression [15]. Consequently, elucidating the precise molecular mechanisms driving PCa pathogenesis remains crucial for developing targeted therapeutic strategies for patients.

THBS2 functions as a calcium‐dependent glycoprotein critically involved in orchestrating fundamental biological processes such as neovascularization, cellular adhesion dynamics, cytoskeletal rearrangement, programmed cell death, and migratory regulation [16]. Under standard physiological conditions, the stabilized N‐terminal regions of THBS2 engage with integrin α4β1 receptors present on microvascular endothelial cells, thereby promoting cell proliferation and facilitating blood vessel formation [17]. Accumulating evidence indicates that THBS2 exhibits dual functions, acting as either a tumor suppressor or a tumor promoter depending on the context of different malignancies. Several studies have highlighted its role as a tumor suppressor, where THBS2 restrains the growth, metastasis, and angiogenesis of various cancers, including myxoid liposarcoma and pancreatic neuroendocrine tumors [18, 19]. Conversely, in other types of cancers such as osteosarcoma, gastric cancer, lung cancer, uveal melanoma, and PCa, THBS2 has been shown to enhance metastatic potential by promoting the secretion of matrix metalloproteinases and activating signaling pathways such as PLC, PKC, c‐Src, NF‐κB, and PI3K [19–23]. The heightened secretory mesenchymal phenotype of breast cancer metastasis‐initiating cells drives effective lung fibroblast activation through THBS2, fostering the development of a pro‐metastatic microenvironment [24]. Furthermore, upregulated THBS2 expression amplifies glycolytic activity in colorectal cancer cells [25]. The diverse roles of THBS2 in various cancers remain unclear; these functions may be associated with the specific cell types responsible for THBS2 production within different tumor environments. This study identified elevated THBS2 expression in PCa tissues through analysis of TCGA database, with subsequent validation in clinical specimens and PCa cell lines. Data demonstrated significant associations between THBS2 upregulation and advanced T, N staging, elevated Gleason score, advanced patient age, and reduced PFI. In vitro, THBS2 overexpression significantly augmented proliferative capacity, stimulated migratory and invasive potential, and inhibited apoptotic processes, whereas THBS2 silencing reversed these malignant phenotypes. These findings indicate that THBS2 functions as an oncogene in PCa, consistent with the report by Chen et al. [23].

As a component of the transforming growth factor‐beta (TGF‐β) superfamily, inhibin subunit beta A (INHBA) mediates diverse physiological processes such as immune modulation, sexual differentiation, stem cell regulation, and coordinated control of cell migration and proliferation [26, 27]. Abnormal expression of INHBA has been observed in several types of cancer, such as ovarian, prostate, breast, and colorectal cancers [28–30]. Li et al. [31] demonstrated that INHBA facilitates neoplastic expansion while driving resistance to PD‐L1 checkpoint inhibition through suppression of interferon‐gamma‐mediated immune surveillance mechanisms. Mechanistic dissection of THBS2 in PCa progression revealed that THBS2 engages in direct physical interaction with INHBA, forming a functional complex that drives oncogenic signaling. This molecular partnership underpins the observed concordance in their expression patterns. Critically, THBS2‐driven enhancement of cellular invasion and proliferation was abrogated upon INHBA suppression, whereas the antitumor effects of THBS2 knockdown were reversed by INHBA restoration. These reciprocal functional perturbations establish INHBA as the nonredundant effector through which THBS2 executes its pro‐malignant functions. Collectively, the THBS2‐INHBA axis emerges as an indispensable regulatory node in PCa pathogenesis.

Through systematic bioinformatic analysis utilizing GO, KEGG, and GSEA, our study revealed significant associations linking elevated THBS2 expression levels to activation of focal adhesion, PI3K/AKT pathway, and EMT processes. FAK, an intracellular non‐receptor tyrosine kinase, serves as a critical regulator of cytoskeletal dynamics and cellular motility [32]. This signaling mediator orchestrates integrin‐mediated signal transduction cascades, enabling downstream activation of PI3K/AKT pathway [33]. FAK/PI3K/AKT signaling cascade serves as a central regulator of cellular proliferation, metastatic progression, and viability maintenance [34]. To gain deeper insight into the molecular mechanisms driving PCa progression via the THBS2‐INHBA axis, we conducted further investigations. Notably, silencing INHBA was able to counteract the THBS2‐induced increases in EMT, as well as the phosphorylation of FAK, PI3K, and AKT. Conversely, overexpressing INHBA diminished the inhibitory effects on the FAK/PI3K/AKT pathway and EMT that resulted from THBS2 knockdown. Most importantly, the functional necessity of this pathway was unequivocally confirmed using the specific FAK inhibitor Y15, which abrogated the THBS2‐driven EMT and malignant progression. Altogether, these findings demonstrate that the interplay between THBS2 and INHBA facilitates the activation of the FAK/PI3K/AKT pathway and EMT in PCa.

## 5. Conclusion

In conclusion, our study demonstrates that in PCa, THBS2 facilitates cell proliferation, migration, invasion, and EMT, while inhibiting apoptosis, by activating the FAK/PI3K/AKT signaling pathway through its association with INHBA. These results highlight the potential of THBS2 not only as a biomarker for PCa diagnosis and prognosis but also as a promising target for therapeutic intervention.

## Ethics Statement

This study received ethical approval from the Institutional Review Board of Tongxiang First People’s Hospital (Approval Number 20213505).

## Consent

All participants provided written informed consent prior to their involvement.

## Conflicts of Interest

The authors declare no conflicts of interest.

## Author Contributions

**Yifeng Yao:** conceptualization, investigation, data curation, writing – original draft, methodology. **Yiyang Guo:** writing – original draft, formal analysis, validation. **Shoulei Liu:** software, visualization, data curation. **Yaojun Li:** supervision, resources, writing review and editing. **Shanmiao Chen:** conceptualization, funding acquisition, methodology, resources, supervision, writing – review and editing. Yifeng Yao and Yiyang Guo contributed equally to this work.

## Funding

This work was supported by the 2022 Zhejiang Province Health and Wellness Science and Technology Plan (Grant 2022KY1277).

## Data Availability

The datasets generated and analyzed during the current study are available upon reasonable request from the corresponding author.
